# Sensitivity and Specificity of *In situ* Proximity Ligation for Protein Interaction Analysis in a Model of Steatohepatitis with Mallory-Denk Bodies

**DOI:** 10.1371/journal.pone.0096690

**Published:** 2014-05-05

**Authors:** Bernhard Zatloukal, Iris Kufferath, Andrea Thueringer, Ulf Landegren, Kurt Zatloukal, Johannes Haybaeck

**Affiliations:** 1 Institute of Pathology, Medical University Graz, Graz, Austria; 2 Department of Immunology, Genetics and Pathology, Uppsala University, Uppsala, Sweden; University of Florida, United States of America

## Abstract

The in situ proximity ligation assay (*is*PLA) is an increasingly used technology for *in situ* detection of protein interactions, post-translational modifications, and spatial relationships of antigens in cells and tissues, in general. In order to test its performance we compared *is*PLA with immunofluorescence microscopy by analyzing protein interactions in cytoplasmic protein aggregates, so-called Mallory Denk bodies (MDBs). These structures represent protein inclusions in hepatocytes typically found in human steatohepatitis and they can be generated in mice by feeding of 3,5-diethoxy-carbonyl-1,4-dihydrocollidine (DDC). We investigated the colocalization of all three key MDB components, namely keratin 8 (K8), keratin 18 (K18), and p62 (sequestosome 1) by *is*PLA and immunofluorescence microscopy. Sensitivity and specificity of *is*PLA was assessed by using *Krt8^−/−^* and *Krt18^−/−^* mice as biological controls, along with a series of technical controls. *is*PLA signal visualization is a robust technology with excellent sensitivity and specificity. The biological relevance of signals generated critically depends on the performance of antibodies used, which requires careful testing of antibodies like in immunofluorescence microscopy. There is a clear advantage of *is*PLA in visualizing protein co-localization, particularly when antigens are present at markedly different concentrations. Furthermore, *is*PLA is superior to confocal microscopy with respect to spatial resolution of colocalizing antigens. Disadvantages compared to immunofluorescence are increased costs and longer duration of the laboratory protocol.

## Introduction

In order to obtain insights in molecular mechanisms of diseases it is increasingly important to investigate not only levels of expression of proteins and their subcellular localization, but also their interactions with other proteins. This is particularly important because proteins may exert different functions depending on their presence in multi-protein complexes and their localization in cellular compartments. Techniques available for investigating the colocalization of proteins within cells and tissues include double-label indirect immune fluorescence (DIIF) microscopy and Förster resonance energy transfer (FRET) microscopy [1]. More recently, a new technique for visualizing proteins and their interactions based on the proximity ligation mechanism, was added to the spectrum of microscopy techniques [2].

The in situ proximity ligation assay (*is*PLA) is a technology that extends the capabilities of traditional immunoassays to also include protein interactions, post-translational modifications, and spatial relationships of antigens in cells and tissues. Protein targets can be readily detected and localized with single molecule resolution and objectively quantified in unmodified cells and tissues. Utilizing only a few cells, even transient or weak interactions are revealed *in situ* and sub-populations of cells can be distinguished. Within hours, results from conventional co-immunoprecipitation and co-localization techniques can be independently confirmed by *is*PLA.

**Figure 1:**
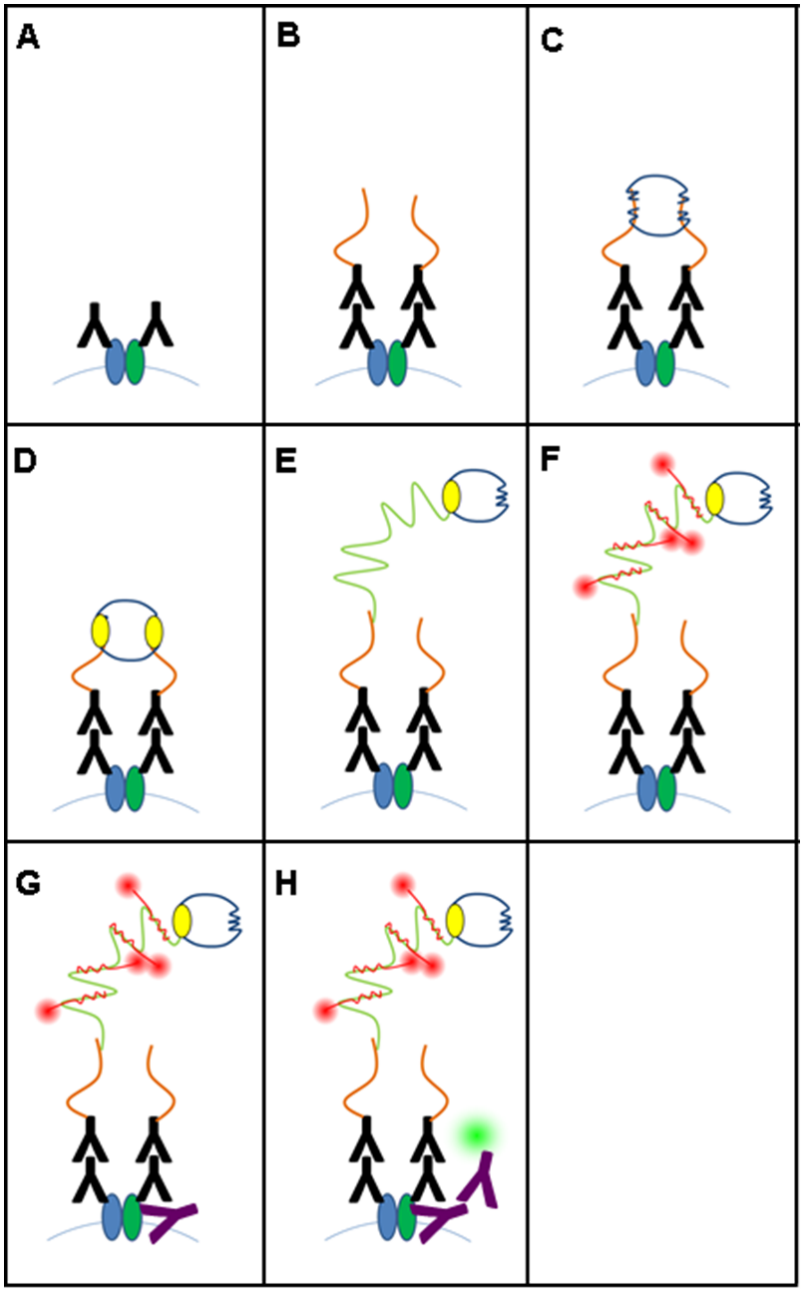
Schematic drawing of different antibody binding and visualization steps of *is*PLA combined with IF. (**A**) Primary ABs of the *is*PLA bind to two different proteins, which are expected to colocalize. (**B**) Secondary ABs conjugated with oligonucleotides bind to the primary ABs (PLA Probe PLUS and PLA Probe MINUS). (**C**) Two more oligonucleotides (blue) hybridize to the two PLA probes. (**D**) Ligase (yellow) joins the two added oligonucleotides to form a closed circle. (**E**) Polymerase (yellow) induces a rolling circle amplification (RCA) using the ligated circle as a template. (**F**) Fluorescence labeled oligonucleotides (red) hybridize to the RCA product. (**G**) Primary ABs of the IF (purple) bind to one of the proteins, which is also targeted by *is*PLA. (**H**) Secondary ABs of the IF bind to the primary ABs of the IF. They are fluorescence labeled in red while the *is*PLA signals are green.

*is*PLA can make use of pairs of primary antibodies raised in different species to recognize the target protein or protein complex of interest. Species-specific secondary antibodies, called PLA probes, each with a unique short DNA strand attached to it, bind the primary antibodies. When the PLA probes are in close proximity, the DNA strands can interact through a subsequent addition of two other circle-forming DNA oligonucleotides. After joining of the two added oligonucleotides by enzymatic ligation, the DNA circles are copied via rolling circle amplification using a polymerase. In the amplification reaction, long DNA strands, each containing several hundred complements of the DNA circle, are formed. These products are easily visualized by microscopy as brightly fluorescent spots using fluorescence-labeled complementary oligonucleotide probes (Fig. 1) [2].

*is*PLA has been used for investigation of a wide variety of disease conditions, for instance for quantification of HER2-protein complexes in breast cancer [3], and for detecting osteopontin-interactions in resectable gastric cancer.

The increasing use of *is*PLA as a tool in pathology prompted us to investigate its sensitivity in detail and specificity in comparison to that of DIIF confocal microscopy. For this purpose, we used experimentally induced multi-protein aggregates in mouse liver as a test system. This model is based on chronic feeding of mice with a diet containing 3,5-diethoxy-carbonyl-1,4-dihydrocollidine (DDC, also known as diethyl 1,4-dihydro-2,4,6-trimethyl-3,5-pyridinedicarboxylate), which leads to the formation of histopathological structures called Mallory-Denk bodies (MDBs) in hepatocytes and to ballooning of the hepatocytes, which are associated with a disruption of the keratin intermediate filament cytoskeleton. MDBs are protein aggregates in the cytoplasm of hepatocytes, which occus in alcoholic and non-alcoholic steatohepatitis (ASH, NASH), copper storage diseases, chronic cholestasis, some types of drug induced liver injuries, and in hepatocytic neoplasms [4]. The MDBs are composed of keratin 8 (K8), keratin 18 (K18), and p62 (sequestosome 1) as main constituents. In this study, we have performed a comprehensive analysis on factors impacting on the visualization of K8, K18 and p62 in MDBs by *is*PLA and confocal microscopy by using this well established experimental animal model. Since for this model mice knocked out for production of K8 and K18 (*krt8^−/−^* and *krt18^−/−^, respectively)* were available [5], [6], we used the knockout mice as biological controls to demonstrate the specificity of *is*PLA in visualization of protein co-localization.

## Materials and Methods

Male Swiss Albino mice (strain Him OF-1; Institute of Laboratory Animal Research, Medical University of Vienna, Himberg, Austria), *Krt8^−/−^*129ola, and *Krt18^−/−^*129ola mice were kept under SPF conditions and fed a standard diet (Ssniff Spezialdiäten GmbH, Soest, Germany). For induction of MDBs, mice were fed a diet containing 0.1% 3,5-diethoxycarbonyl-1,4-dihydrocollidine (DDC; Sigma-Aldrich, Vienna, Austria) for 12 weeks [6]. Mice were killed by cervical dislocation and liver tissues were snap frozen in methyl-butane cooled to the temperature of liquid nitrogen. The animal experiments have been approved by the Austrian Federal Ministry of Science and Research, Division of Genetic Engineering and Animal Experiments (Vienna, Austria) (BMBWF-66.010/0060-II/3b/2013).

For DIIF and *is*PLA, mouse livers tissue sections (2 µm) were fixed with acetone for 10 min at −20°C. After air drying, slides were incubated with the DUOLINK Blocking Stock reagent (Olink, Uppsala, Sweden) diluted 1∶5 in Antibody Diluent for 30 min at 37°C. Slides were incubated with primary ABs for *is*PLA (mouse-anti-Keratin 8 [Ks 8.7 Progen] 1∶50 or mouse-anti-Keratin18 [18.4 Progen] 1∶50, and guinea pig anti-p62 [p62CT] 1∶200 in Antibody Diluent) for 30 min at room temperature in a humidified chamber (both primary antibodies were applied together) and then washed 3×5 min in TBS-T. Thereafter slides were incubated with PLA Probes from DUOLINK anti-mouse PLA PLUS and anti-guinea pig PLA MINUS diluted 1∶10 in Antibody Diluent for 30 min at 37°C and washed 2×5 min at 37°C in TBS-T. For hybridization, slides were incubated with DUOLINK hybridization stock diluted 1∶5 in AB diluent for 15 min at 37°C and washed 1×1 min in TBS-T at 37°C. For the ligation, slides were incubated with DUOLINK ligation reagent (Ligation Stock 1∶5 in AB diluent plus ligase [1∶40]) for 15 min at 37°C and washed 2×2 min in TBS-T at 37°C. For signal amplification, DUOLINK Amplification Stock was diluted 1∶5 in AB diluent and polymerase (1∶80) was added and slides were incubated for 90 min at 37°C followed by 2×2 min washings in TBS-T at 37°C. For the detection, DUOLINK Detection Stock (Texas red; 613 nm) was diluted 1∶5 in AB diluent and slides were incubated for 60 min at 37°C. For the final washing, four sequential washing steps were performed with 2×SSC, 1×SSC, 0.2×SSC and 0.02×SSC for 2 min each at room temperature.

For the additional DIIF staining, rabbit-anti-K8+18 (50K160; for review on MDB-reacting antibodies see [7] or rat-anti-K8 (TROMA1 tissue culture supernatant, neat) were used as primary antibodies. Primary antibodies were incubated for 30 min at room temperature, followed by 3×5 min washings in PBS and incubation with secondary antibodies [Alexa 488-conjugated anti-rabbit (Invitrogen), diluted 1∶100 or Alexa 488-conjugated anti-rat (Invitrogen), diluted 1∶100] for 30 min at room temperature. After 3×5 min washes in PBS slides were rinsed in 100% ethanol, air-dried and mounted in Mowiol^R^ (Sigma-Aldrich). Fluorescence signals from *is*PLA and DIIF were detected by Zeiss LSM 510 META scanning laser confocal microscope. Excitation wavelength for Alexa 488 was 488 nm (Argon Laser), and for the *is*PLA reagent 594 nm (HeNe Laser) (analysis software: ZEN 2009) was used. A summary of all reaction steps for the combination of *is*PLA and DIIF is shown in Figure 1.

As controls for specificity of *is*PLA a series of technical and biological negative controls were performed. Technical controls were based on the omission of first antibodies (to exclude cross-reactivity of secondary antibodies or non-specific PLA-signal generation). Biological controls involved knock-out mice for two of the antigens present in MDBs. In this case all primary and secondary antibodies were applied, however because of the absence of one of the antigens detected in PLA, no PLA-related signals should be detected. Sensitivity of PLA was assessed by comparison with single and double-label immunofluorescence analyzed by confocal microscopy. A summary of all controls performed is shown in Table 1.

**Table 1:** Technical controls performed on mice with and without DDC treatment.

Sample	AB PLA m-a-K8	AB PLA gp-a-p62	PLA Probe PLUS	PLA Probe MINUS	PLA Detect. Kit	AB IIF rat-a-K8	Alexa 488 (a-rat)	Rhod. (a-gp)	Result
Standard diet	x	x	x	x	x	x	x		*PLA and IIF positive*
Standard diet	x	x	x	x	x				*only PLA positive*
Standard diet						x	x		*only IIF positive*
DDC diet	x	x	x	x	x	x	x		*PLA and IIF positive*
DDC diet	x	x	x	x	x				*only PLA positive*
DDC diet						x	x		*only IIF positive*
DDC diet	x		x	x	x		x		*PLA and IIF negative*
DDC diet		x	x	x	x		x		*PLA and IIF negative*
DDC diet		x				x	x	x	*double IIF positive*

In order to complement the biological controls (knockout mice) we performed a number of technical controls to make sure that no cross reactions between the different reagents might lead to false results.

## Results

**Figure 2:**
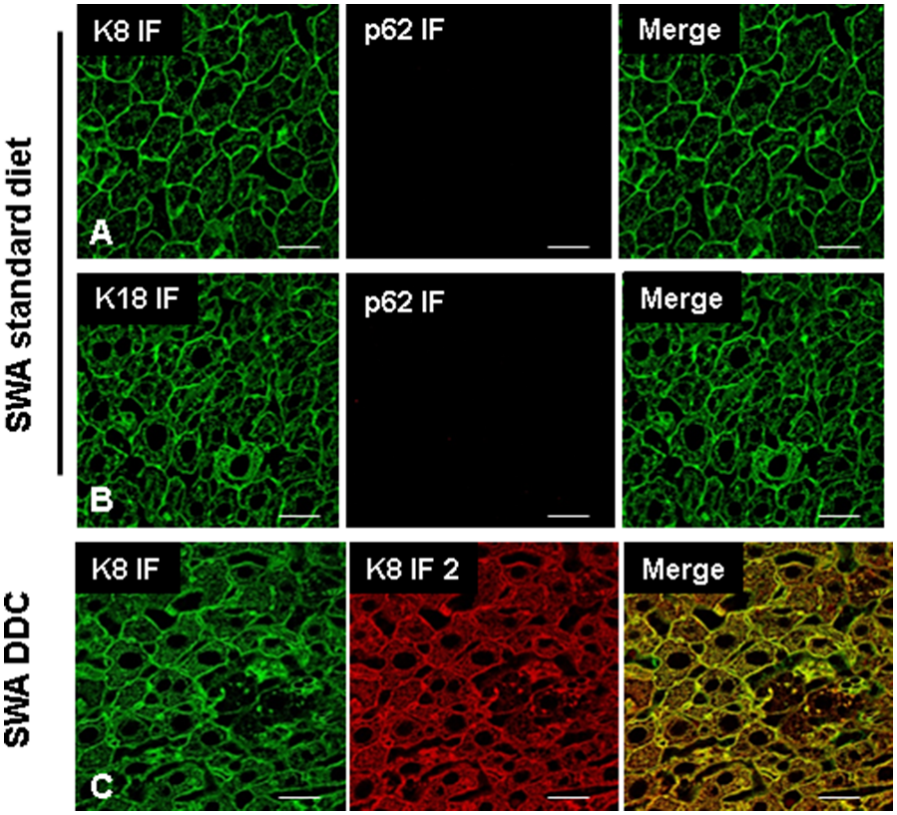
Testing of ABs to keratin and p62 in mouse liver and demonstration of equivalent binding properties of ABs used in IF and *is*PLA. IF staining of normal mouse liver tissues (Swiss Albino, SWA fed a standard diet) with antibodies to K8 (green), p62 (red) (**A**) and for K18 (green) and p62 (red) (**B**). Image (**C**) shows that the two different ABs directed to K8 (rat-anti-K8 [Troma I] and mouse-anti-K8 [Ks 8.7 Progen]) used for *is*PLA and IF or DIIF recognize identical structures in liver from a DDC fed SWA mouse. Scale bars: 20 µm.

In a first approach, the reactivity of the antibodies was tested. All antibodies (ABs) used in this study were applied for single and double-label indirect immunofluorescence (IF) microscopy in different sequential orders. Additionally, the primary ABs were tested by using different fluorochrome-labeled secondary ABs in various combinations to ensure equivalent reactivity (data not shown). Irrespective of the experimental setup identical results were observed for antibodies raised in different species but directed to the same antigen (i.e., K8 and K18, respectively) (Fig. 2).

This was essential because two different AB pairs, raised in different species (e.g., mouse and rat), were used in the combined *is*PLA and IF staining. Only if it was ensured that both ABs, namely the keratin AB used in IF and the keratin AB used in *is*PLA had identical staining properties, could differences between IF and *is*PLA be evaluated. Otherwise any observed differences between IF and *is*PLA could depend on different staining properties of the primary ABs.

**Figure 3:**
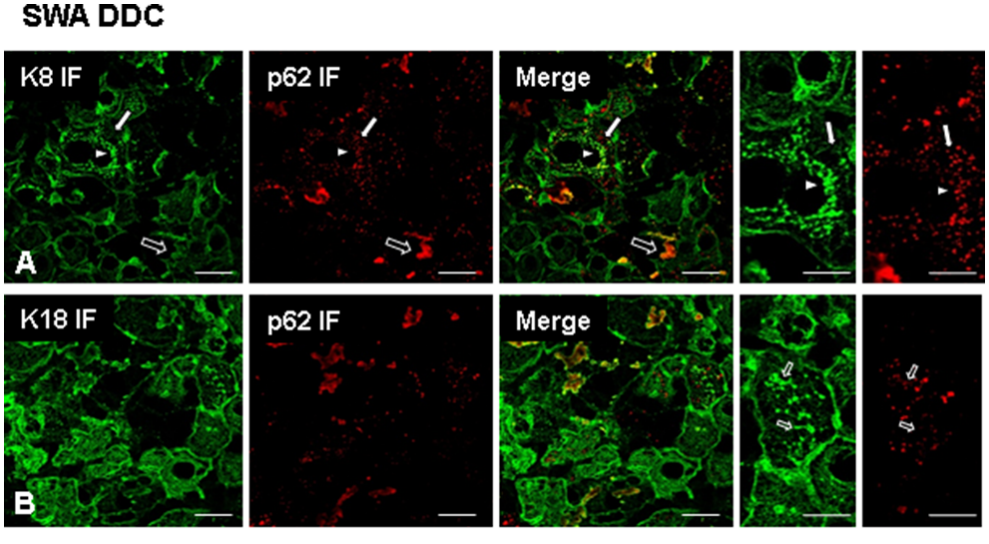
Testing of ABs to keratin and p62 in mouse liver cells after DDC treatment. Immunofluorescence stain on SWA mouse livers after 12 weeks DDC treatment using ABs to K8 (green), p62 (red) (**A**), and K18 (green), p62 (red) ABs (**B**). For image (**A**), note the presence of small (arrowhead) and large (empty arrow) MDBs. Additionally, small granules positive for p62 only can be observed (arrow). For image (**B**), note that small MDB-like keratin aggregates can be observed that are negative for p62 (empty arrow). Scale bars: 20 µm.

Figure 2 shows a double–label IF of wild type Swiss Albino mice before and after DDC treatment using the optimal AB combinations as identified in the pre-testing phase. Normal mouse livers did not show any protein aggregations positive for p62, but exhibited a filamentous architecture of the keratin intermediate filament cytoskeleton in the hepatocytes. After DDC treatment ballooned hepatocytes were present with a disrupted keratin cytoskeleton and MDBs involving K8, K18 and p62 were observed (Fig. 3). In addition to the classical MDB, which is positive for keratin and p62, small p62 positive but K8/K18 negative structures were present [8]. However, the nature of these structures has not been characterized yet. Furthermore, small keratin positive MDB-like aggregates were seen in which p62 was only weakly present or even undetectable. It was not possible to draw clear conclusions about p62 positivity in these structures, since the observed results were influenced by the threshold and amplification settings for the different fluorescence channels during confocal microscopy (Fig. 3 B).

**Figure 4:**
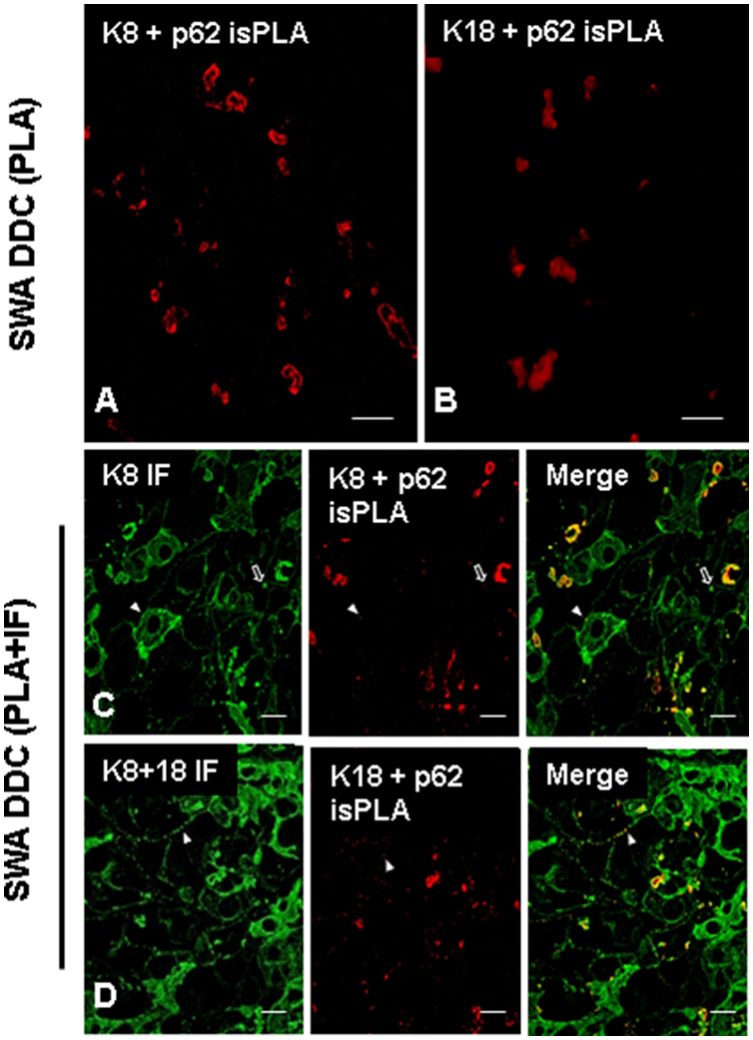
*is*PLA for 12 week-DDC treated mice. Images (**A, B**) show *is*PLA signal (red) using ABs to p62 and K8 (**A**) and p62 and K18 (**B**). (**C**) shows a combined visualization of p62 and K8 in *is*PLA (red) and K8 in IF (green). (**D**) shows a combined visualization of p62 and K18 in *is*PLA (red) and K18 IF (green). In images (**C**) and (**D**) note that even very small *is*PLA positive structures are visible (arrowhead), demonstrating the high sensitivity of *is*PLA. However, there were also larger MDB-like keratin aggregates that are negative for *is*PLA stain (empty arrow), suggesting the absence of p62. Scale bars: 20 µm.

The same liver used in the experiment shown in Fig. 3, and the same combinations of ABs were applied for *is*PLA alone, and *is*PLA in combination with IF (Fig. 4). For the *is*PLA, two ABs (m-a-K8 and gp-a-p62) were used to detect protein aggregations in which p62 and K8 co-localized. A third AB (rat-a-K8) was applied on the same tissue sample as the primary AB for the IF. Since *is*PLA only gives a positive signal if both components (keratin, p62) were present, every MDB detected by the *is*PLA would also be expected to be positive for keratin 8 in the IF stain. The combined *is*PLA and IF stain confirmed the presence of K8 in every *is*PLA positive MDB. Moreover, it was notable that *is*PLA detected the smallest MDBs, visible by DIIF. However, some keratin aggregates resembling MDBs were negative for p62 both in *is*PLA and IF (Fig. 3 and Fig. 4). In summary, the experiments demonstrated that the sensitivity of *is*PLA is at least as high as the sensitivity of DIIF. As technical negative controls for the *is*PLA the first ABs to p62 and keratin, respectively, were omitted. All negative controls constantly showed negative *is*PLA signals (negative controls not shown).

**Figure 5:**
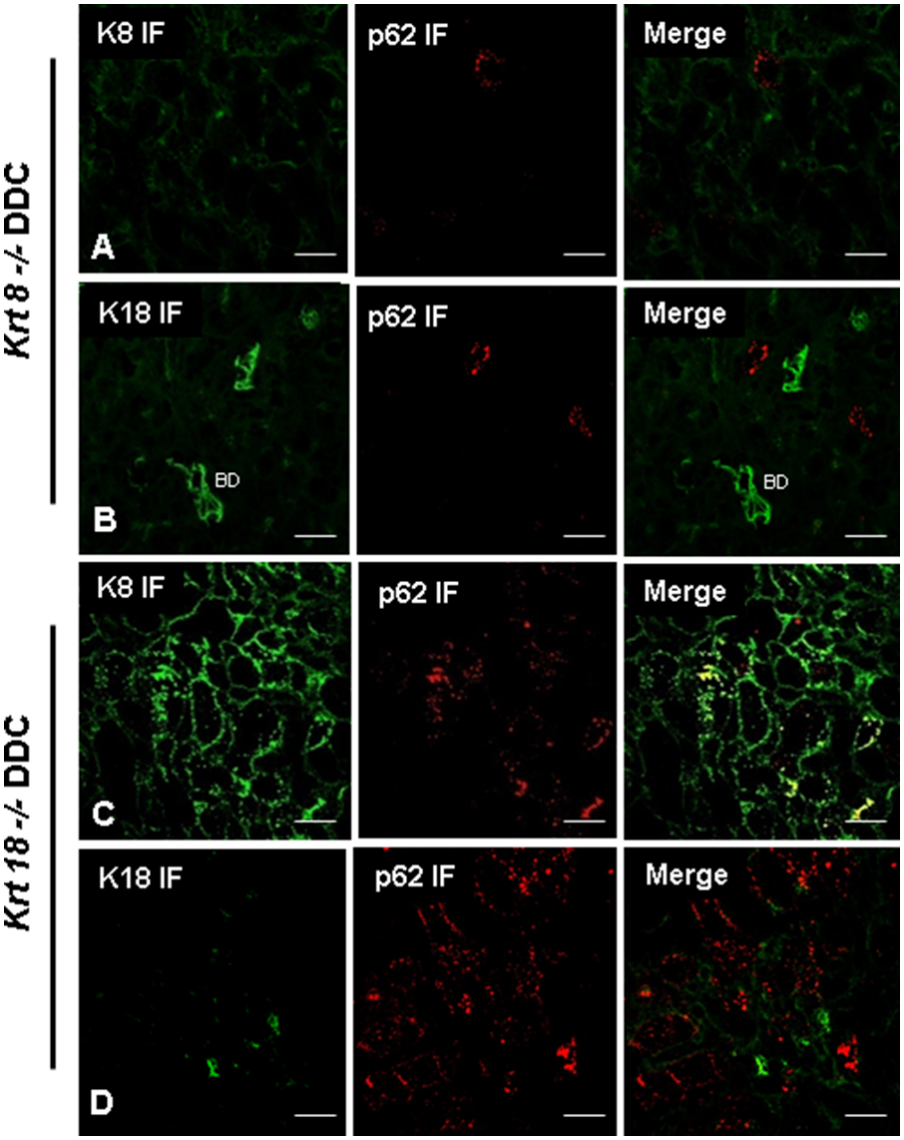
Demonstration of keratin and p62 in DDC treated *krt8^−/−^* and *krt18^−/−^* mice. IF staining on *krt8^−/−^* (**A, B**) and *krt18^−/−^* (**C, D**) mouse livers after 12 weeks DDC treatment. Images (**A**) and (**C**) show the localization of K8 (green) and p62 (red). Images (**B**) and (**D**) show the localization of K18 (red) and p62 (red). A positive colocalization of p62 and K8 in *krt18^−/−^* mice was observed, which indicates presence of MDBs. In contrast *krt8^−/−^* mice did not develop MDBs after DDC feeding, therefore neither K18 nor p62 accumulated. ABs to K18 show positivity in bile ducts (BD) in *krt8^−/−^* mice (**B**). The green reaction in image (**D**) relates to plasma cells. Scale bars: 20 µm.

**Figure 6:**
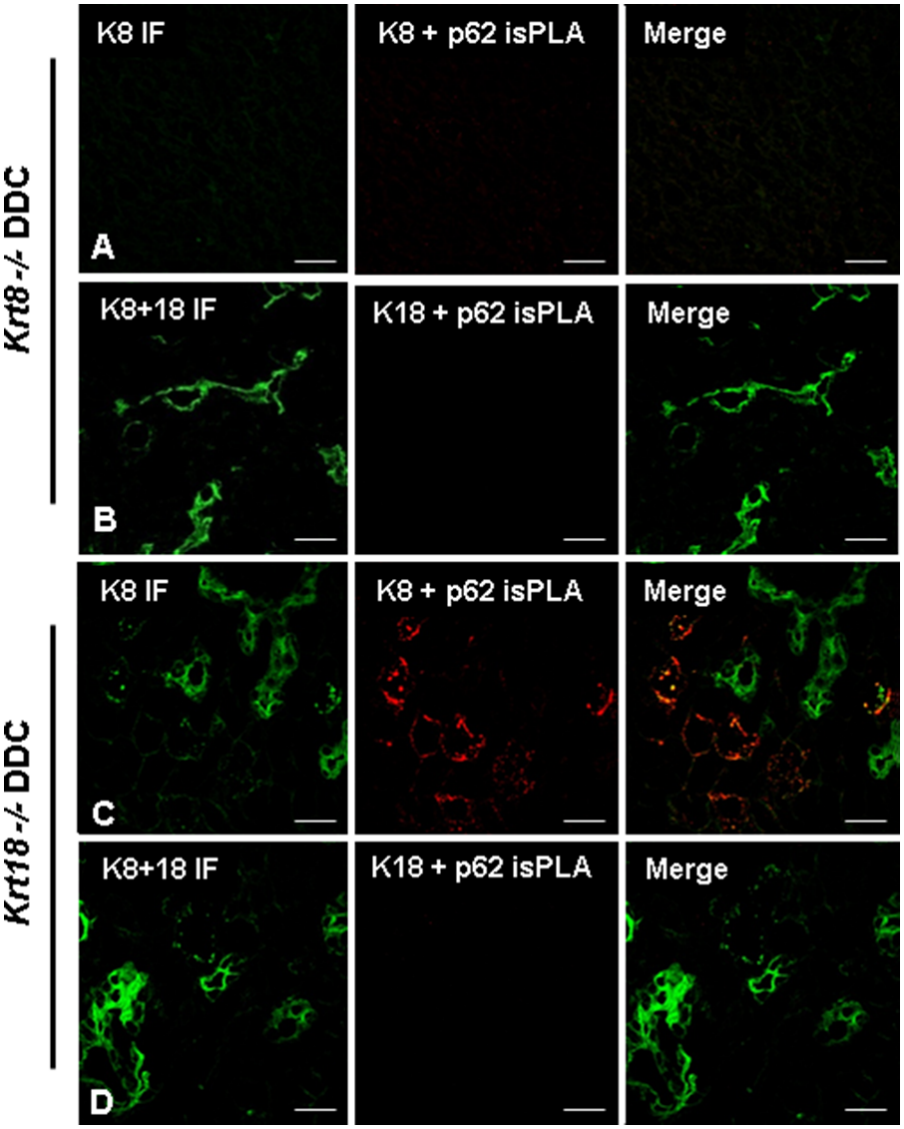
*is*PLA and IF for 12 week-DDC treated *krt8^−/−^* and *krt18^−/−^* mice. (**A, B**) *krt8^−/−^* mouse liver. (**A**) Combined *is*PLA (K8+ p62, red) and IF (K8, green). (**B**) Combined *is*PLA (K18+ p62, red) and IF (K8+ K18, green). (**C, D**) *krt18^−/−^* mouse liver. (**C**) combined *is*PLA (K8+ p62, red) and IF (K8, green). (**D**) Combined *is*PLA (K18+ p62, red) and IF (K8+ K18, green). Note that *krt8* ^−/−^ do not form MDBs after DDC feeding and there is no *is*PLA positive signal. In DDC fed *krt18^−/−^* mice *is*PLA yielded only positive signals with K8 ABs demonstrating specificity of the whole *is*PLA system. Scale bars: 20 µm.

**Figure 7:**
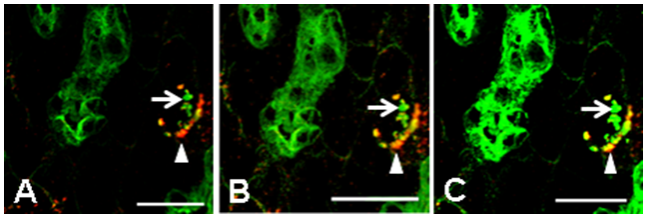
Influence of channel amplification on demonstration of antigen colocalization in *is*PLA and IF in 12 week-DDC treated *krt18^−/−^* mice. (**A, B, C**) Show different levels of digital amplification of the green channel (IF for K8) and constant amplification of the red channel showing *is*PLA for K8 and p62. Arrows indicate a MDB-like aggregate that was constantly positive for K8 but negative for p62. Arrowheads indicate MDBs which were positive in *is*PLA for p62 and K8 but IF showed only presence of K8 (yellow merged signal) in *is*PLA positive MDBs after maximal amplification of the green channel. Scale bars: 20 µm.

Reactivity of ABs used was further assessed in *Krt8* and *Krt18* knock-out mice. In both knock-out mice hepatocytes were negative for keratin intermediate filament cytoskeleton because, in the absence of one partner (e.g., K8) the other still expressed partner (e.g., K18 in *Krt8−/−* mice) is unstable and does not form intermediate filaments (Fig. 5). In the absence of a polymerization partner, K8 or K18 is quickly degraded. The only structures that were stained positive for the expressed keratin partner in mice fed a standard diet were bile ducts since in bile duct epithelia additional keratins are expressed that form intermediate filaments [8], [9]. DIIF microscopy of DDC-fed *krt8* knock-out mice using ABs to p62 and K8, respectively, demonstrated the absence of MDBs as previously published [8]. *krt8^−/−^* mice showed small p62 positive cytoplasmic structures which were negative for keratin. In contrast, *krt18^−/−^* mice developed MDBs that were composed of K8 and p62 [6] (Fig. 5). These mice were then tested by applying combined *is*PLA and IF for p62 and K8 or K18 (Figs. 6 and 7). The *krt8^−/−^* mice were negative both in the K18+p62 and K8+p62 *is*PLA, which underlines the absence of keratin in the small p62 positive structures observed in DIIF (Fig. 3). Furthermore immunofluorescence demonstrated keratin positive aggregates with typical MDB structure which were negative in *is*PLA. This is in line with previous results demonstrating by double-label immunofluorescence microscopy that some MDB-like aggregates were p62-negative [7]. Thus our *is*PLA results support these previous findings with an independent technology. In the *krt18^−/−^* mice *is*PLA revealed positive MDBs only in case of using K8 and p62 ABs as primary ABs for the *is*PLA. In contrast, the *is*PLA stain with ABs to K18 and p62 was negative. In *krt18^−/−^* mice all components of the *is*PLA (K18 and p62 ABs, amplification system, detection system) were applied but no reaction products were observed. This is consistent with the fact that only one of the two antigens was present, namely p62, while K18 was absent, demonstrating that *is*PLA is highly specific. Furthermore, these data demonstrated that no cross reactivity occurred between the ABs used for *is*PLA and those used in IF (in the DIIF a polyclonal rabbit-AB to K8+18 was used, whereas for *is*PLA a mouse monoclonal AB to K18 was used). This underlined the species specificity of the ABs used in the *is*PLA system and that there is no cross reactivity between *is*PLA and DIIF.

## Discussion

In this study we have performed a comprehensive investigation of clinically relevant morphological alterations by using *is*PLA. We have chosen a mouse model for steatohepatitis and MDB formation for testing various methodologies because the main molecular components and interaction partners of MDBs, namely K8, K18, and p62, are well known and characterized, and a broad spectrum of antibodies and models were available. In order to demonstrate the sensitivity and specificity of *is*PLA we have performed technical as well as biological controls, by including gene knock-out mice for the MDB components K8 and K18.

We applied *is*PLA in combination with IF and compared results with DIIF. Signals of *is*PLA and IF or DIIF were analyzed by confocal microscopy. For these studies it was essential to ensure that ABs used for *is*PLA and DIIF had equivalent staining properties, and that secondary ABs did not cross-react with other primary ABs. Since performance of *is*PLA critically relies on the performance of the ABs used, the scheme of technical controls performed in this study can be recommended for establishing *is*PLA essentially for any application (see Table 1). Under the prerequisite of proper performance of ABs, results obtained demonstrated that *is*PLA is a robust, sensitive and specific tool for investigating the relationship between proteins and their association with each other in tissues. In general, findings of *is*PLA were similar to those of DIIF. However, *is*PLA led to much clearer results when one antigen was more abundant than the second one because *is*PLA only yields a signal in case of antigen colocalization (e.g., small MDB-related keratin aggregates in the presence of the keratin intermediate filament cytoskeleton). In contrast, results of DIIF analyzed by confocal microscopy depended on the settings of the different fluorescent channels. Therefore we recommend *is*PLA as a preferred technique for visualization of protein colocalization in small structures, particularly when larger, strongly positively stained structures are present as well (this situation causes problems in setting the appropriate background thresholds in DIIF). For instance, because of these properties of *is*PLA our analysis provided evidence that p62 was lacking in some MDB-like keratin aggregates. This new observation may shed new light on the role of p62 in MDB pathogenesis.

Furthermore, since *is*PLA signals depend on the molecularly defined maximal distance between the antigens, *is*PLA provides better defined resolution for protein colocalization than DIIF *is*PLA is estimated to permit identification of pairs of epitopes located within less than approximately 40 nm. This is a 5-fold higher resolution compared to DIIF, which allows the detection of distinct signals with a distance of approximately 0.2 µm (along the x- and y-axes) due to limitations of standard light microscopy. Since the optical section thickness of confocal microscopy is in the range of 0.5 µm (z-axis), *is*PLA is of particular advantage for demonstrating colocalization of antigens along the z-axis. However, the resolution of *is*PLA is lower than that of FRET which detects molecular interactions at a distance of even below 0.5 nm [10].

In recent years other techniques like Array Tomography with High-Resolution Three-Dimensional Immunofluorescence have also been developed. This is a volumetric microscopy method based on physical serial sectioning where ultrathin sections are cut, stained and imaged. The resulting two-dimensional images are then further reconstructed by using computational methods in order to generate three-dimensional volume images, suitable for quantitative analysis [11]. This method is more difficult to realize than *is*PLA.

In conclusion, we found that the *is*PLA provides more accurate results compared to DIIF because of the signal specificity, the better spatial resolution and the independence of the results and exposure time and digital amplification of microscope channels used (Fig. 7; pros and cons of *is*PLA are summarized in Table 2). As the function of proteins critically depends on their interactions with other molecules and the localization of multi component complexes in specific subcellular components, techniques for *in situ* detection of protein complexes become important. Furthermore, personalized medicine application does not only depend on the expression of the drug target but also on the activation of the target-related pathway, which is characterized by specific interactions between cellular components. Therefore application of *is*PLA might become increasingly relevant for molecular diagnosis and research requiring a carefully controlled and robust protocol, for which examples and practical guidance is provided in this study.

**Table 2:** Comparison of DIIF microscopy and *is*PLA for analyzing the colocalization of antigens.

Feature	Double-label IF	PLA
Sensitivity	++	+++
Specificity	++	+++
Dynamic range*	+	+++
Spatial resolution	++	+++
Protocol time	+++	+
Costs	+++	+

Relative advantages are indicated in + to +++.

*: dynamic range is particularly important for visualizing colocalization of high and low abundance antigens, which also impacts on overall sensitivity and specificity.
